# Does air pollution inhibit digital finance? Evidence from Chinese prefecture-level cities

**DOI:** 10.1371/journal.pone.0294314

**Published:** 2023-11-20

**Authors:** Liya Zheng, Tao Cen, Qiaoyun Wu

**Affiliations:** 1 College of International Economics and Trade, Ningbo University of Finance and Economics, Ningbo, China; 2 Ningbo Philosophy and Social Science Key Research Base "Research Base on Digital Economy Innovation and Linkage with Hub Free Trade Zones", Ningbo, China; 3 College of Wealth Management, Ningbo University of Finance and Economics, Ningbo, China; Sichuan University, CHINA

## Abstract

Air pollution poses significant health and economic challenges globally and specifically affecting China. Although air pollution has been associated with decreased productivity and biases in decision-making, its effect on the development of digital finance has received limited attention in the literature. By employing city-level data from China covering the period from 2013 to 2020, this research examines the impact of air pollution on digital finance. The results show that deteriorating air quality has a negligible impact on digitalization, whereas it has a negative impact on financial inclusion, measured by usage and coverage metrics. The negative impact on financial inclusion is more noticeable in economically weaker and less developed urban areas and low R&D than in developed areas and economically robust cities. The mechanism analysis shows that air pollution reduces human capital quality, resulting in a decline in financial inclusivity. These findings have significant policy implications, underscoring the necessity for approaches that simultaneously tackle air pollution and foster financial innovation.

## 1. Introduction

The increasing influence of air pollution on environmental sustainability and the well-being of human populations has emerged as a prominent area of interest for researchers and decision-makers. Furthermore, growing research suggests that this pollution may have far-reaching consequences for mental health as well as physical health. In addition to declining physical and mental health, exposure to these pollutants contributes to adverse emotional reactions and impairs cognitive abilities. In addition to the health implications, air pollution has economic consequences [1–4]. At the individual level, it has the potential to intensify behavioral biases among investors, impedes the ability to make sound decisions [5], and disrupt productivity in the workplace [6–8]. On a macro level, it poses risks to countries’ manufacturing productivity. Given these multifaceted impacts, it’s essential to understand the broader ramifications of air pollution on economic structures, particularly financial inclusion and digitalization. Financial inclusion, which ensures that individuals and businesses can access valuable and affordable financial products and services, can be particularly vulnerable.

In today’s global society, environmental challenges have far-reaching implications that affect human health, economies, and technological progress, among other domains. Although the immediate health consequences of air pollution are well-known, the far-reaching implications for financial systems and the advancement of technology have received less attention. In recent empirical investigations, the relationship between air pollution and diverse aspects of economic performance has been extensively examined [9–12]. Ref. [13] discovered a correlation between air pollution and the trading activities of individual investors in China, indicating that trade performance deteriorated on cloudier days. Ref. [14] demonstrated that extended exposure to air pollution significantly impairs cognitive abilities, with an increased susceptibility observed among seniors. The cognitive deterioration resulting from pollution has immediate financial consequences in addition to health implications. Further expanding on this notion, Ref. [15] studied the effect of air pollution on the development of corporate human capital and discovered that elevated pollution levels have an adverse effect on the capacity of organizations to retain proficient personnel, consequently impeding the productivity and value of the organization. Although the studies mentioned above highlight the complex financial consequences of air pollution, there is a lack of studies on the role of financial digitalization.

Digital finance has received extensive attention in recent years as it reduces financial costs and lowers the threshold for financial services. Digital finance enhances financial services’ coverage, depth, and breadth [16, 17], benefiting specific populations such as rural residents, low-income individuals, and small and medium-sized enterprises. Research on digital finance has focused on investigating the economic effect of digital finance. However, there are few studies on the factors influencing the development of digital finance. Some scholars have explored these factors from the perspective of financial exclusion, such as Ref. [18], who discovered that countries with severe financial exclusion also exhibit low levels of digital finance. At the same time, variables such as residents’ education level, income, and age influence the development of digital finance in countries like Peru and Argentina [19, 20]. Moreover, government intervention is vital in developing digital finance [21]. Previous research studies have linked air pollution to organizational performance, productivity, and other economic indicators. However, there is a lack of research that specifically investigates the impact of air pollution on the digitization of financial services, a vital and swiftly advancing aspect in modern economies.

This paper aims to address the gaps in the existing research by exploring the impact of air pollution on the development of digital finance. The primary aims of this study are twofold: First, to determine the degree to which air pollution affects the progress of digital finance and whether this impact differs during its different phases of development. Second, this study aims to clarify the fundamental mechanisms that mediate this correlation. Furthermore, we examine the distinct impacts that urban heterogeneity has on digital finance and air pollution. In this study, we also explore the influence of air pollution on human capital quality. This research study significantly contributes to the existing literature in different ways. First, this paper innovatively links air pollution to digital finance, enriching the literature on air pollution and its economic consequences. Second, based on quasi-natural experiments, this paper uses instrumental variables and discontinuity regression methods to identify the causal relationship between air pollution and digital finance. Compared to traditional estimation methods, the regression discontinuity approach exhibits features similar to those of randomized experiments and provides enhanced control over biases arising from endogeneity [22]. Moreover, the conclusions of this paper have vital theoretical and practical implications for the policymaker to control air pollution and develop the inclusiveness of digital technology. The significant consequences of air pollution on digital finance will offer vital knowledge for informing policy decisions and promoting financial innovation.

## 2. Theoretical foundation and research hypothesis

### 2.1 Air pollution and digital finance

Air pollution, widely recognized as a significant public health issue, has effects that go beyond just physical well-being. The notion that health depreciation can be influenced by environmental factors, such as air pollution, was first proposed by Grossman in 1972 [23]. Similarly, several research studies have shown that air pollution has a determinantal influence on human cognition and behavior. For example, Ref. [14] confirmed that extended exposure to air pollution impairs cognitive performance on verbal and mathematical assessments. Moreover, this consequence becomes more pronounced with an individual’s age, presenting considerable difficulties for individuals depending on cognitive abilities for routine activities and crucial judgments. The consequences of this decline in cognitive performance extend beyond individual responsibilities and encompass financial conduct and the economy. According to Ref. [13], investor trading performance deteriorates on days with severe air pollution. The trend indicates that commerce quality deteriorated as the severity of air pollution increased. It has been hypothesized that air pollution may cause investors to exhibit certain behavioral biases, highlighting the potential concealed costs of air pollution on stock market dynamics. However, air pollution’s effects extend beyond individual cognition and behavior.

The corporate sector also feels the cumulative effects. A compelling tendency was observed among experienced professionals to pursue employment opportunities in less polluting regions when confronted with severe air pollution in their present locations. The phenomenon of this migration, commonly referred to as the ’brain drain,’ denotes a decline in the quantity and caliber of human resources in areas with pollution, potentially leading to an adverse impact on the operations and output of businesses [15]. Moreover, when businesses face the difficulties presented by declining air quality, they modify their corporate conduct. The findings of Ref. [24] research suggest that businesses, especially those in high-energy consumption sectors, tend to increase their corporate social responsibility (CSR) efforts in response to deteriorating air quality. Companies’ adaptive measures to mitigate the negative externalities of their operations in the face of the tension between economic and environmental sustainability are reflected in this increase in CSR. As the effects of air pollution are investigated in more depth, its influence on digital finance becomes more evident. Numerous variables influence the development of digital finance, including infrastructure, the caliber of human capital, and the cognitive capacities of its user population. Based on the established evidence regarding the detrimental effects of air pollution on cognitive functions and its indirect repercussions on the quality of human capital, it is possible to posit a negative correlation between air pollution and the advancement of digital finance. The rationale for this hypothesis is rooted in the potential deterioration of cognitive functions observed in groups exposed to air pollution. When considering the brain outflow phenomenon, which can potentially diminish the quality of human capital in contaminated areas, the development and implementation of innovative financial tools in those regions may face a plausible barrier. Drawing from the preceding discussion, the present study puts forward the following hypothesis:

*Hypothesis 1*: *There is an association between air pollution and digital finance*.

China exhibits significant regional disparities in economic development, which manifests in infrastructural capacities, technological progress, and talent acquisition. Greater degrees of urbanization confer a competitive advantage on cities, enabling them to attract highly skilled individuals and accelerate the transfer of innovative ideas. Consequently, this drives the advancement of digital finance [25]. On the contrary, economically disadvantaged areas frequently face a scarcity of financial resources allocated to investigate and advance digital financial infrastructure. This scarcity may be exacerbated in areas where air quality is substandard. The adverse effect of air pollution on digital finance growth may be exacerbated by the simultaneous challenges of environmental degradation and economic underdevelopment [26]. It is worth stating that organizations functioning in regions troubled with inadequate air quality frequently employ monetary motivations, such as increasing managerial remuneration, to retain their most skilled personnel. In light of the discussion above, this paper proposes the following hypothesis to enrich the literature further.

*Hypothesis 2a*: *Urbanization plays a moderating role between air pollution and digital finance*.
*Hypothesis 2b*: *The impact of air pollution on digital finance varies with the city’s economic development and R&D spending*.
*Hypothesis 2c*: *The impact of air pollution on digital finance varies with salary level*.

## 3. Methods

### 3.1 Variable setting and data description

This study uses a sample of 337 prefecture-level in China from 2013 to 2020. Digital finance is measured using DFI (digital finance index), DIF_COVER (coverage of digital finance), DIF_USAGE (degree of Usage of digital finance), and DIF_DIGIT (the degree of digitalization). DFI represents the total development level of digital finance. The three digital finance sub-indices include DIF_COVER, which represents coverage breadth, and DIF_USAGE, which represents usage depth. Furthermore, DIF_DIGIT represents the digitization degree. The data for the above variables has been taken from the Digital Finance Research Center of Peking University [17, 27]. Air pollution is measured by the annual average concentration of PM10, collected from the China Environmental Yearbook and the official website of the Ministry of Ecology and Environment. In the robustness test, air pollution is proxied by PM2.5 and AQI (Air Quality Index), collected from the China Research Data Services Platform and the official website of the Ministry of Ecology and Environment.

In this study, we’ve used lnINVER as the instrumental variable, which represents the logarithm of the number of days a city experiences thermal inversion plus one. The data originates from the National Aeronautics and Space Administration (NASA) database. First, we matched each city to the NASA grid layer database based on its latitude and longitude. A thermal inversion is identified on a given day when the atmospheric temperature of the city’s corresponding second horizontal layer is higher than that of the first layer.

In the regression discontinuity analysis, the policy variable is GAP. GAP is a dummy variable. China is exogenously divided into north and South based on the Huai River line. GAP equals "1" if the city is located in northern China and otherwise "0". In this study, we have taken different moderating variables, and the moderating variables include urbanization level, urban R&D level, and economic development level. The data for these variables are sourced from China Population Statistics Yearbook and China Statistical Yearbook. The data on the management salary level of firms and human capital quality are from the CSMAR database. Following previous studies [28], the following control variables are added: financial development level of the city (Finance Level), GDP, and industrial output value (Manufacturer). The specific variable names and their descriptive statistics are shown in Table 1.

**Table 1:** Descriptive statistics.

Variables	Variable meaning	Mean	Std	Min	Max	Obs
DIF	Total development level of Digital Finance	5.55	0.504	2.79	6.07	10980
DIF_COVER	The Coverage Breadth of Digital Finance	5.40	0.366	1.91	5.79	10980
DIF_USAGE	Usage Depth of Digital Finance	5.38	0.404	2.52	5.86	10980
DIF_DIGIT	The degree of digitalization	5.39	0.550	1.29	6.00	10980
PM10	Annual average PM10 concentration	0.082	0.038	0.027	0.348	10980
PM2.5	Annual average PM2.5 concentration	0.052	0.025	0.012	0.198	10980
AQI	Annual average of air quality index	79.9	30.7	31.2	500	10980
Inversion	The number of days with temperature inversion per year	241	95.1	29.0	366	10980
lnInversion	log (Inversion+1)	5.38	0.497	3.40	5.90	10980
Financial Level	The ratio of a city’s financial institution loans to its GDP at the end of the year.	1711	1137	19.1	3801	10980
ln GDP	Log of GDP	1.69	0.615	0.289	5.80	10980
ln Manufacturer	Log of the city’s annual industrial output value	18.5	1.07	13.215	19.7	10980
Population	Log of the total urban population	6.48	0.700	3.37	8.14	10980
Older Support	Elderly dependency ratio	0.123	0.021	0.00	0.23	10980
Older	The proportion of population aged 65 and above	0.089	0.016	0.038	0.16	10980

### 3.2 Research design

#### 3.2.1 Regression discontinuity design (RDD)

Based on the study of Ref. [28, 29], this study has taken the Huaihe River line as a breakpoint to develop a fuzzy regression discontinuity model. The heating policy varies between China’s southern and northern regions, exogenously separated by the Huai River line [28]. From November to March each year, the government provides centralized heating in the northern region. In contrast, the southern region lacks such heating due to resource and budget constraints. In winter, China’s central heating predominantly relies on coal, which, when burned, releases significant amounts of sulfur dioxide and suspended particulates into the atmosphere. As a result of this heating policy, air pollution is more pronounced in the northern region compared to the southern region. In the absence of the Huai River policy, we would expect a gradual variation in air quality based on geographic latitude. However, because of this policy, there’s a noticeable discontinuity in air pollution levels at the Huai River boundary. If we observe a similar discontinuity in digital finance near the Huai River line, we can infer that other factors influencing digital finance don’t have such a breakpoint at this line. Afterward, we can infer that the heating policy causes a discontinuity in air quality, leading to differences in digital finance development between northern and southern cities near the Huai River line. In this paper, referring to Ref. [30], a two-stage fuzzy regression discontinuity model is set as follows:

$$PM10_{i,t}=\vartheta_{0}+\vartheta_{1}{GAP}_{i}+\vartheta_{2}f(L_{i})+\vartheta_{3}{Gap}_{i}*f(L_{i})+\sum CV_{i.t}+\nu_{i.t}$$ (1)

$${Digital\;Finance}_{i,t}=\tau_{0}+\tau_{1}\hat{{PM10}_{i,t}}+\tau_{2}f(L_{i})+\tau_{3}GAP*f(L_{i})+\sum CV_{i,t}+\omega_{i,t}$$ (2)

Model (1) is the first-stage regression model, with GAP as a dummy variable. GAP equals one if the city is located north of the Huai River line and zero otherwise. The model includes the latitude-driving variable *L*_*i*_ and the polynomial *f*(*L*_*i*_) of the difference between the latitude of the Huai River dividing line to control the linear or nonlinear impact of the latitude difference with the dividing line on air pollution. This impact may differ between the north and South of the Huai River line. Therefore, the interaction term of *GAP*_*i* and *f*(*L*_*i*_) is added to the model. Model (2) is the second-stage regression. In model (1) and model (2), year and city fixed effects are added.

#### 3.2.2 Ordinary least squares regression (OLS)

In this study, we also employed the ordinary least squares regression (OLS) estimation method to explore the impact of air pollution on digital finance. Following previous studies [12, 31, 32], the regression model is given as:

$${Digital\;Finane}_{i,t}=\alpha_{0}+\alpha_{1}PM10_{i,t}+\sum CV_{i,t}+\sum Year+\varepsilon_{i,t}$$ (3)

In this model, Digital Finance includes DIF, DIF_COVER, DIF_USAGE, and DIF_DIGIT. The core independent variable is PM10, the PM10 coefficient α_1_ is the focus of this paper. If α_1_ is negative, this indicates that air pollution inhibits the development of digital finance and ∑CV_i,t_ shows the control variables.

#### 3.2.3 Instrumental variable

To mitigate endogeneity concerns, we use an instrumental variable strategy. Following the study of Ref. [33], we have taken thermal inversion (lnINVER) as an instrumental variable. lnINVER is the logarithm of the number of days with the city’s thermal inversion per year. Commonly, the atmospheric temperature is negatively correlated with the altitude. If the atmospheric temperature rises with increasing altitude, thermal inversion happens [34]. The thermal inversion layer can suppress convection in the atmospheric boundary layer, hindering the dispersion of smoke, impurities, and harmful gases resulting in severe air pollution [33]. Thermal inversion is associated with haze, sandstorms, and other phenomena [35]. Thus, thermal inversion is related to air pollution but has not been linked with digital finance. Therefore, it is a potential and reasonable instrumental variable for air pollution. Following the study of Ref. [36], we employed the two-stage least squares regression model given as follows:

$$PM10_{i,t}=\phi_{0}+\phi_{1}ln\;{INVER}_{i,t}+\sum CV_{i,t}+\nu_{i.t}$$ (4)

$${Digital\;Finance}_{i,t}=\theta_{0}+\theta_{1}\hat{{PM10}_{i,t}}+\sum CV_{i,t}+\psi_{i,t}$$ (5)

## 4. Results and discussion

The analytical aspects of this study are explained in this section. The graphical analysis offers a preliminary visual insight, allowing for an intuitive grasp of data trends. After the initial, we employed the Regression Discontinuity Design (RDD) to explore the primary linkage between the variables. In this study, we also carried out the second stage RDD analysis to assess the causal effect of the treatment on the dependent variable. Additionally, in this section, we provide the results from the OLS regression analysis and the instrumental variable.

**Fig 1:**
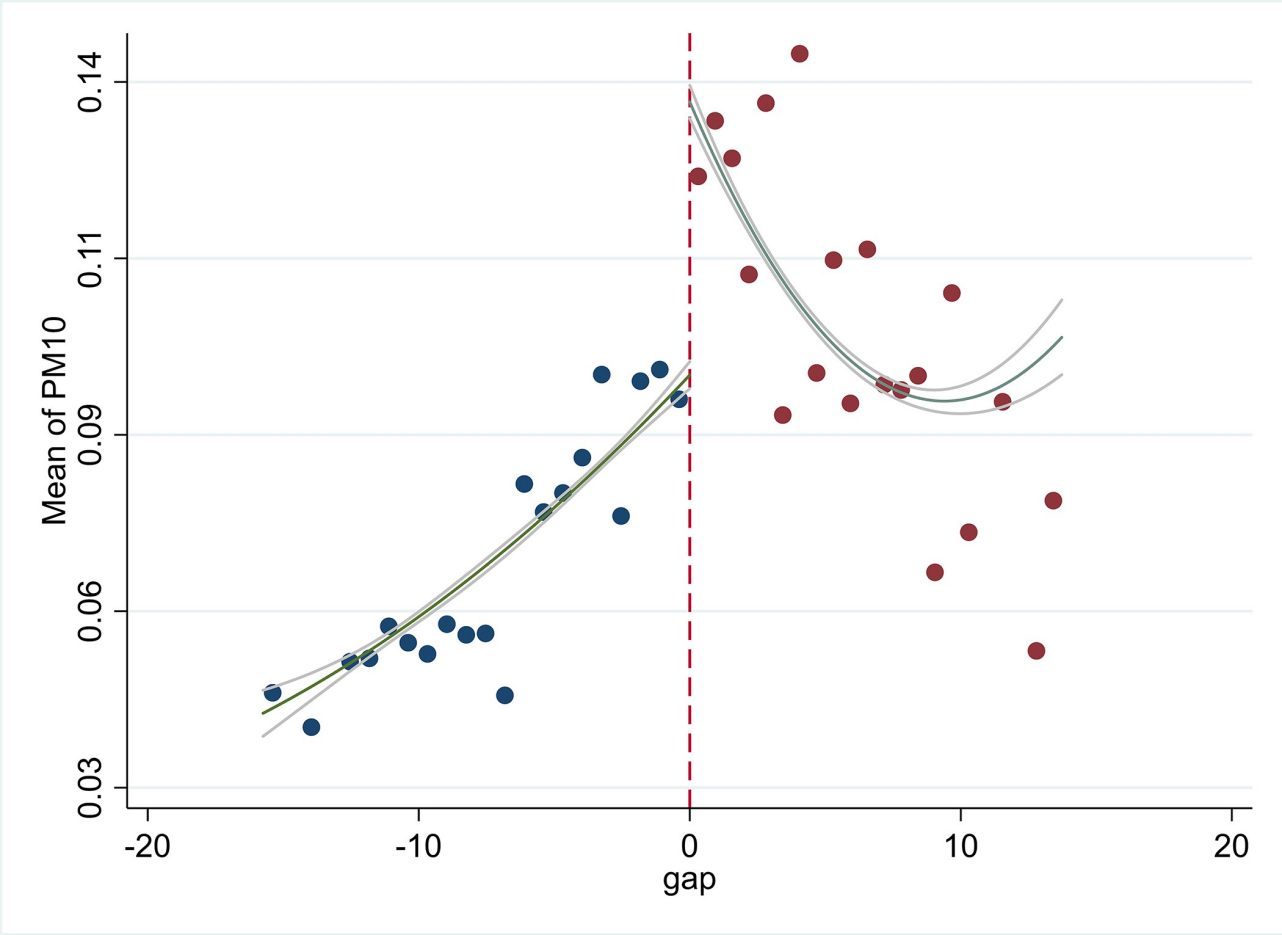
Breakpoint graph of PM10.

**Fig 2:**
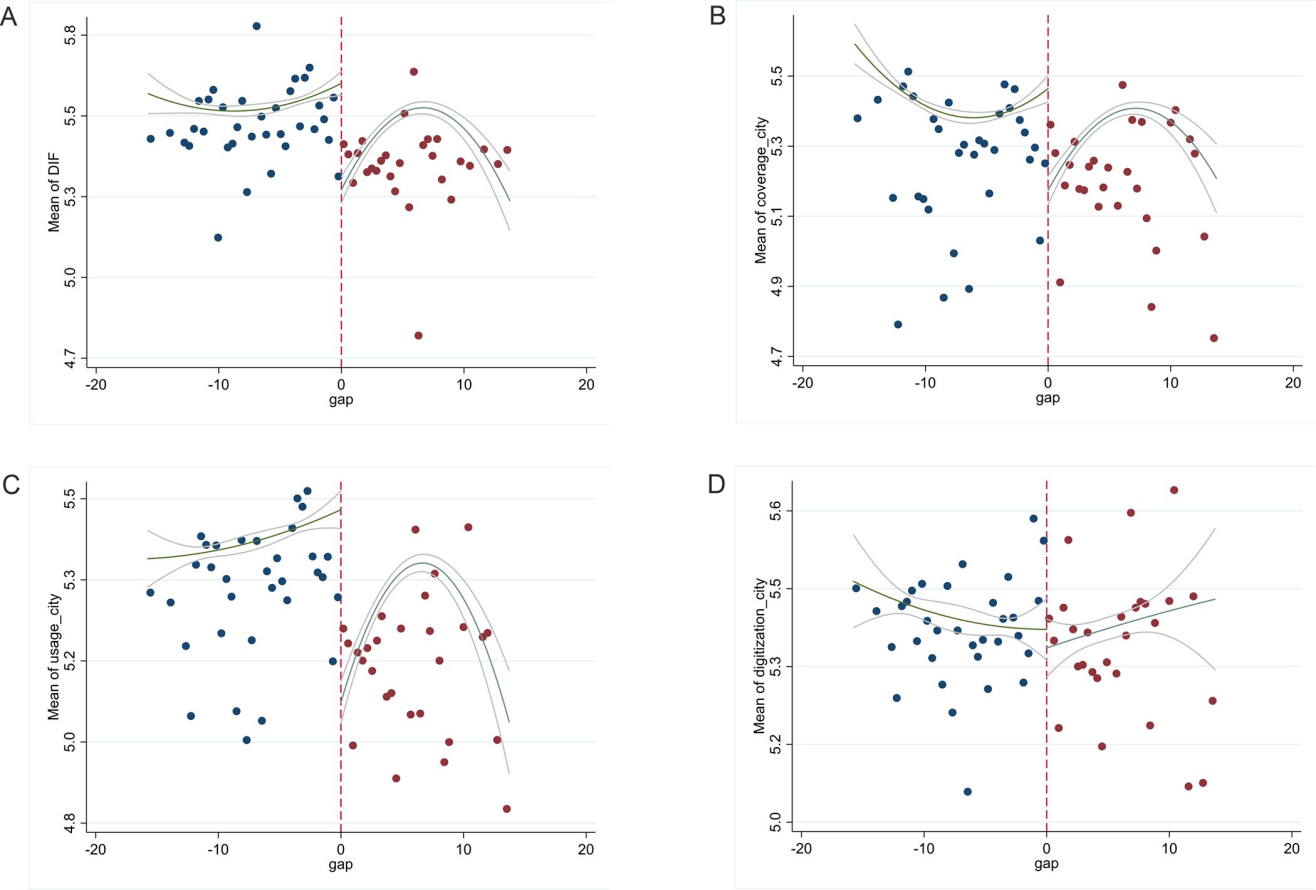
Breakpoint graph of digital finance. (A) Breakpoint graph of DIF. (B) Breakpoint graph of DIF_COVER. (C) Breakpoint graph of DIF_USAGE. (D) Breakpoint graph of DIF_DIGIT.

Figs 1 and 2 show the variations in PM10 concentration and the digital finance index for sample cities based on the Huai River line from 2013 to 2020. The dots depict the average value within a one-degree latitude range for each city. Fig 1 reveals a noticeable spike in PM10 concentration close to the Huai River boundary. Fig 2A–2C respectively demonstrates that the digital finance total index (DIF), coverage breadth (DIF_COVER), and usage depth (DIF_USAGE) all display a distinct rise near this demarcation. However, Fig 2D indicates no significant shift in the digital finance index (DIF_DIGIT) at this breakpoint.

### 4.1 The first-stage regression results of RDD

Table 2 presents the first-stage regression results of the RDD analysis. Columns (1) and (2) give the results for 5490 city-year data situated within a ±10° latitude range of the Huai River line. In Column (1), without the inclusion of control variables, the coefficient of GAP is notably positive. When the control variables, year, and city fixed effects are integrated in Column (2), the GAP coefficient stands at 0.03, at a 1% level of significance. This indicates that the average PM10 concentration in northern cities exceeds that of southern cities by 0.03 mg/m^3^. In Column (3), which encompasses a full-sample regression, the GAP coefficient remains positively significant at the 1% level. The test for a weak instrument variable rejects the null hypothesis of GAP being weak as an instrumental role.

**Table 2:** The first stage regression result of the RDD.

	(1)	(2)	(3)
samples	±10°	±10°	Whole samples
GAP	0.014***	0.030***	0.026***
	(0.000)	(0.000)	(0.000)
lnManufacturer		-0.083***	-0.058***
		(0.000)	(0.000)
FinancialLevel		0.018	0.012
		(0.140)	(0.247)
lnGDP		0.090***	0.163***
		(0.000)	(0.000)
constant	0.0239***	0.1327***	0.1832***
	(0.000)	(0.000)	(0.000)
CV	No	Yes	Yes
City	Yes	Yes	Yes
Year	Yes	Yes	Yes
N	5490	5490	10980
R^2^	0.835	0.851	0.698
F-statistic in the first stage	1297	1317	1029

Note: p-values in parentheses, * p < 0.1

** p < 0.05

*** p < 0.01.

### 4.2 The second-stage regression results of RDD

Table 3 reports the second-stage regression results based on 5490 city-year samples within the ±10° latitude range of the Huai River line. For the overall digital finance index (DIF), the coefficient of PM10 is negative and significant at the 5% level. For both coverage breadth and usage depth, the coefficient of PM10 is negative, showing significance at the 10% level. However, for the digital index (DIF_DIGIT), the coefficient of PM10 isn’t significant. The RDD findings underscore that air pollution considerably hinders the development of digital finance, with usage depth being the most affected aspect. These results align with the findings of Ref. [14, 27], which posits that disparities in digital finance between southern and northern China predominantly manifest in usage depth. Moreover, the digital finance index seems less influenced. Given that the digital index primarily centers around aspects like mobility, practicality, creditworthiness, and convenience and is heavily reliant on modern communication technologies and associated infrastructure, its flexibility is comprehensible. The widespread adoption of smartphones ensures that digital finance achieves a more expansive geographical reach, allowing it to more effectively navigate the limitations of traditional financial and physical networks.

**Table 3:** The second stage regression result of the RDD.

	±10°			
	DIF	DIF_COVER	DIF_USAGE	DIF_DIGIT
$\hat{PM10}$	-0.099**	-0.057*	-0.061*	0.022
	(0.034)	(0.071)	(0.075)	(0.674)
lnManufacturer	-0.003	0.029***	0.072***	-0.063***
	(0.858)	(0.009)	(0.000)	(0.001)
FinancialLevel	0.024**	0.117***	0.070***	0.021*
	(0.025)	(0.000)	(0.000)	(0.097)
lnGDP	0.094***	0.047***	0.013	0.063***
	(0.000)	(0.000)	(0.301)	(0.001)
constant	-0.0875**	-0.0453*	-0.0549*	-0.1057**
	(0.044)	(0.090)	(0.085)	(0.042)
CV	Yes	Yes	Yes	Yes
Year	Yes	Yes	Yes	Yes
City	Yes	Yes	Yes	Yes
N	5490	5490	5490	5490

Note: p-values in parentheses, * p < 0.1

** p < 0.05

*** p < 0.01.

### 4.3 OLS and instrumental variable analysis

Table 4 gives the OLS regression results examining the impact of air pollution on digital finance. The findings indicate that for every 0.1mg/m^3^ increase in PM10, DIF decreases by 0.106, DIF_COVER by 0.087, DIF_USAGE by 0.097, and DIF_DIGIT by 0.034. These results emphasize that air pollution substantially hinders the growth of digital finance, with DIF_USAGE representing the frequency of user accounts being the most adversely affected dimension. In cities suffering from intense air pollution, there may be a demographic shift characterized by an outflow of residents, particularly younger individuals frequently utilizing platforms like Alipay and WeChat Pay. This potentially results in diminished utilization of digital financial instruments. Conversely, DIF_Digit’s resilience to the effects of air pollution can be attributed to its composition, which is grounded in aspects such as mobility, creditworthiness, affordability, and convenience. As highlighted by Ref. [27], DIF_digit is less geographically constrained, explaining its relative stability amidst escalating pollution levels.

**Table 4:** OLS regression results.

	(1)	(2)	(3)	(4)
	DIF	DIF_COVER	DIF_USAGE	DIF_DIGIT
PM10	-1.062***	-0.874***	-0.972***	-0.340*
	(0.000)	(0.000)	(0.000)	(0.052)
lnManufacturer	0.013	0.065***	0.051***	0.006
	(0.379)	(0.000)	(0.000)	(0.690)
FinancialLevel	0.010	0.110***	0.050***	0.020**
	(0.360)	(0.000)	(0.000)	(0.037)
lnGDP	0.055***	0.004	0.017	-0.002
	(0.000)	(0.721)	(0.154)	(0.879)
Constant	-1.745***	-4.040***	-3.156***	-0.443
	(0.000)	(0.000)	(0.000)	(0.103)
Year Fixed	Yes	Yes	Yes	Yes
Obs	10980	10978	10979	10979
R^2^	0.058	0.119	0.081	0.016

p-values in parentheses, * p < 0.1

** p < 0.05

*** p < 0.01

Table 5 presents the results from the IV regression analysis. Columns (1) and (2) display the first-stage regression results, with the coefficient of log of inversion (lnINVER) being significantly positive at the 1% level. Based on the f-statistic, we reject the null hypothesis, suggesting that lnINVER is a robust instrumental variable. Columns (3) through (6) indicate that the coefficients for the digital finance development index, as well as its three distinct dimensions, are all significantly negative at the 5% level. This aligns with the findings from the OLS regression analysis.

**Table 5:** Instrumental regression results.

	The first stage regression		The second stage regression			
	(1)	(2)	(3)	(4)	(5)	(6)
	PM10	PM10	DIF	DIF_COVER	DIF_USAGE	DIF_DIGIT
lnINVER	0.036***	0.036***				
	(0.000)	(0.000)				
$\hat{PM10}$			-0.925***	-1.871***	-1.223***	-0.458*
			(0.001)	(0.000)	(0.000)	(0.097)
_cons	0.064***	0.044***	4.846***	4.838***	4.799***	5.289***
lnManufacturer		0.010***	0.014	0.063***	0.051***	0.005
		(0.000)	(0.355)	(0.000)	(0.000)	(0.705)
FinancialLevel		0.004***	0.011	0.108***	0.050***	0.019**
		(0.000)	(0.328)	(0.000)	(0.000)	(0.042)
lnGDP		0.012***	0.055***	0.004	0.017	-0.002
		(0.000)	(0.000)	(0.683)	(0.155)	(0.882)
CV	No	Yes	Yes	Yes	Yes	Yes
Year	Yes	Yes	Yes	Yes	Yes	Yes
City	Yes	Yes	Yes	Yes	Yes	Yes
N	10980	10980	10980	10978	10979	10979
R^2^	0.632	0.635	0.061	0.110	0.078	0.016

Note: p-values in parentheses, * p < 0.1

** p < 0.05

*** p < 0.01.

The usage depth, denoted as DIF_USAGE, is constructed using six indicators: payment, insurance, money market fund, investment, credit, and loan. We employ these indicators as the dependent variables in the instrumental variable regression. Table 6 details the second-stage regression outcomes for these indicators. As evident from the coefficients in columns (1), (4), (5), and (6), air pollution exerts a significant negative influence on the majority of these segmentation indicators.

**Table 6:** Regression results of segmented indicators for the DIF_Useage.

	(1)	(2)	(3)	(4)	(5)	(6)
	Payment	Insurance	Money market fund	Investment	Credit	Loan
$\hat{PM10}$	-2.072***	0.149	-0.623	-3.350***	-6.049***	-4.512***
	(0.000)	(0.779)	(0.379)	(0.000)	(0.000)	(0.000)
lnManufacturer	15.036***	22.136***	7.877***	-6.803***	8.740***	10.375**
	(0.000)	(0.000)	(0.002)	(0.001)	(0.000)	(0.039)
FinancialLevel	16.140***	24.599***	8.215***	7.804***	3.706***	19.832***
	(0.000)	(0.000)	(0.000)	(0.000)	(0.000)	(0.000)
lnGDP	0.479	1.432	5.834**	27.872***	-2.299**	11.394**
	(0.842)	(0.748)	(0.024)	(0.000)	(0.036)	(0.030)
_cons	-3.653***	-3.228***	-3.510***	-1.359***	-3.472***	-2.296***
	(0.000)	(0.000)	(0.000)	(0.000)	(0.000)	(0.000)
Year	Yes	Yes	Yes	Yes	Yes	Yes
City	Yes	Yes	Yes	Yes	Yes	Yes
N	10980	10980	10980	10980	10980	10980
R^2^	0.098	0.065	0.081	0.135	0.095	0.076

Note: p-values in parentheses, * p < 0.1

** p < 0.05

*** p < 0.01.

### 4.4 Robustness and validity test for RDD

#### 4.4.1 Robustness test of RDD

To validate the robustness of our findings, we conducted the discontinuity regression analysis using four different bandwidths, selecting latitude ranges of ±7°, ±9°, ±11°, and ±12° along the Huai River line. Table 7 displays the second-stage discontinuity regression outcomes for these varying bandwidths. Across all four bandwidths, the influence of PM10 on the digital finance index consistently emerges as significantly negative at the 5% level. For usage depth, the coefficient of PM10 is significantly negative at the 10% level. However, PM10 doesn’t significantly impact the digitization index. These findings align with our initial results, confirming the robustness of the conclusions across different bandwidths.

**Table 7:** The second stage regression results for different broadband.

Panel A ±7° and ±9° latitude range								
	±7°				±9°			
	DIF	DIF_COVER	DIF_USAGE	DIF_DIF_DIGIT	DIF	DIF_COVER	DIF_USAGE	DIF_DIF_DIGIT
$\hat{PM10}$	-0.099**	-0.056*	-0.071**	0.017	-0.097**	-0.054*	-0.062*	0.019
	(0.040)	(0.080)	(0.048)	(0.762)	(0.039)	(0.084)	(0.070)	(0.719)
lnManufacturer	0.000	0.027**	0.080***	-0.067***	0.007	0.037***	0.082***	-0.059***
	(0.987)	(0.027)	(0.000)	(0.001)	(0.679)	(0.002)	(0.000)	(0.003)
FinancialLevel	0.045***	0.118***	0.083***	0.025*	0.043***	0.121***	0.081***	0.028**
	(0.000)	(0.000)	(0.000)	(0.066)	(0.000)	(0.000)	(0.000)	(0.032)
lnGDP	0.085***	0.050***	0.002	0.066***	0.084***	0.045***	0.005	0.060***
	(0.000)	(0.000)	(0.856)	(0.001)	(0.000)	(0.000)	(0.679)	(0.002)
Year	Yes	Yes	Yes	Yes	Year	Yes	Yes	Yes
City	Yes	Yes	Yes	Yes	City	Yes	Yes	Yes
N	7157	7157	7157	7157	7466	7466	7466	7466
Panel B ±11° and ±12° latitude range								
	±11°				±12°			
	DIF	DIF_COVER	DIF_USAGE	DIF_DIGIT	DIF	DIF_COVER	DIF_USAGE	DIF_DIGIT
$\hat{PM10}$	-0.101**	-0.056*	-0.065*	0.010	-0.102**	-0.056*	-0.068*	0.004
	(0.032)	(0.072)	(0.061)	(0.852)	(0.035)	(0.083)	(0.055)	(0.944)
lnManufacturer	0.003	0.041***	0.059***	-0.050***	-0.019	0.057***	0.035***	-0.039**
	(0.846)	(0.000)	(0.000)	(0.004)	(0.174)	(0.000)	(0.001)	(0.011)
FinancialLevel	0.020*	0.112***	0.062***	0.017	0.017*	0.116***	0.056***	0.026**
	(0.053)	(0.000)	(0.000)	(0.152)	(0.081)	(0.000)	(0.000)	(0.015)
lnGDP	0.082***	0.037***	0.020*	0.051***	0.093***	0.020**	0.037***	0.042***
	(0.000)	(0.000)	(0.089)	(0.003)	(0.000)	(0.039)	(0.001)	(0.006)
CV	Yes	Yes	Yes	Yes	Yes	Yes	Yes	Yes
Year	Yes	Yes	Yes	Yes	Yes	Yes	Yes	Yes
City	Yes	Yes	Yes	Yes	Yes	Yes	Yes	Yes
N	9011	9011	9011	9011	10807	10807	10807	10807

Note: p-values in parentheses, * p < 0.1

** p < 0.05

*** p < 0.01.

#### 4.4.2 Validity test of RDD

**Fig 3:**
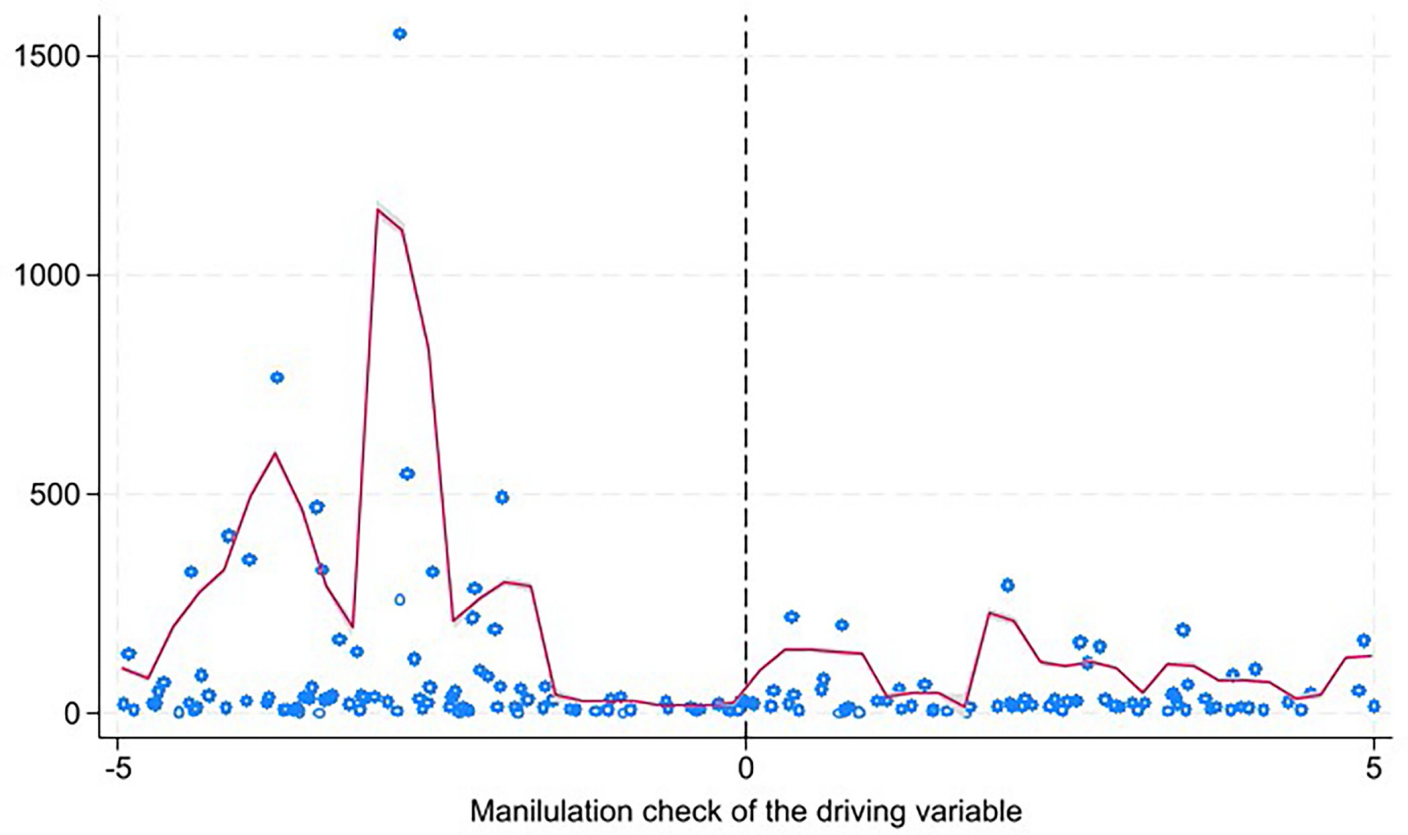
Manipulation check of the driving variable.

RDD analysis requires two fundamental assumptions. Firstly, apart from the selected variables, all other variables potentially influencing the development of digital finance should exhibit smooth transitions at the breakpoint. To address this, we examined the continuity of other variables in Table 2 close to the breakpoint. We defined a dummy variable, NORTH: it is set to 1 if a location is beyond the Huai River line and 0 otherwise. By regressing the latitude difference between each city’s position and the Huai River line against their cross-product, we found that the coefficients of NORTH are not significant. This suggests a continuous transition of the characteristic variables at the breakpoint, thereby affirming the appropriateness of the RDD employed in this study. The second essential assumption for RDD is that there shouldn’t be any potential manipulation of the variables at the regression breakpoints. The advantage of discontinuity regression is its ability to pinpoint selected samples accurately, ensuring the continuity of the implementing variables [37, 38]. The establishment of the Huai River line wasn’t driven by administrative or economic objectives. Instead, this demarcation primarily ascended due to variations in average geographical temperatures. Numerous studies have confirmed that the implementing variable isn’t subject to artificial interference. Fig 3 graphically presents the distribution of the implementing variable’s density function, displaying continuity at the breakpoints, which rules out the possibility of human-induced manipulation of the implementing variables.

### 4.5 Other robustness checks: Replacing core explanatory variables

We substituted the primary explanatory variable, PM10, with PM2.5 and AQI. The PM2.5 data is sourced from the air pollution raster data published by Columbia University’s International Earth Science Information Network Center. Using this data minimizes potential human interference, making it a more reliable measure than air pollution indices derived from ground-level monitoring. Table 8 displays the regression outcomes for the instrumental variables. The coefficients of lnINVER are significantly positive at the 1% level, while the regression coefficients for PM2.5 and AQI are negative at the same significance level. This reinforces the robustness of our findings.

**Table 8:** IV regression results using PM2.5 and AQI as explained variable.

Panel A	(1)	(2)	(3)	(4)	(5)	(6)
	$\hat{PM2.5}$	$\hat{PM2.5}$	DIF	DIF_COVER	DIF_USAGE	DIF_DIGIT
lnINVER	0.013***	0.012***				
	(0.000)	(0.000)				
$\hat{PM2.5}$			-0.976***	-1.289***	-0.924***	-0.651**
			(0.001)	(0.000)	(0.000)	(0.024)
_cons	0.025***	-0.015	4.673***	3.991***	4.117***	5.193***
	(0.000)	(0.448)	(0.000)	(0.000)	(0.000)	(0.000)
lnManufacturer		1.380	-0.010	0.046***	0.036***	-0.016
		(0.408)	(0.498)	(0.000)	(0.001)	(0.233)
FinancialLevel		1.115**	0.014	0.110***	0.050***	0.025***
		(0.033)	(0.173)	(0.000)	(0.000)	(0.006)
lnGDP		-8.423	0.079***	0.025**	0.033***	0.017
		(0.111)	(0.000)	(0.020)	(0.004)	(0.258)
Year	Yes	Yes	Yes	Yes	Yes	Yes
City	Yes	Yes	Yes	Yes	Yes	Yes
N	10980	10980	10980	10980	10980	10980
R^2^	0.863	0.864	0.055	0.119	0.078	0.016
Panel B	(1)	(2)	(3)	(4)	(5)	(6)
	$\hat{AQI}$	$\hat{AQI}$	DIF	DIF_COVER	DIF_USAGE	DIF_DIGIT
lnINVER	15.724***	15.633***				
	(0.000)	(0.000)				
$\hat{AQI}$			-0.001***	-0.001***	-0.001***	-0.000**
			(0.001)	(0.000)	(0.000)	(0.044)
lnManufacturer		0.003***	-0.008	0.048***	0.038***	-0.016
		(0.005)	(0.552)	(0.000)	(0.001)	(0.250)
FinancialLevel		0.001**	0.013	0.109***	0.050***	0.024***
		(0.022)	(0.206)	(0.000)	(0.000)	(0.008)
lnGDP		-0.003**	0.079***	0.025**	0.033***	0.018
		(0.035)	(0.000)	(0.020)	(0.005)	(0.249)
_cons	46.611***	94.065**	4.692***	4.016***	4.152***	5.194***
	(0.000)	(0.013)	(0.000)	(0.000)	(0.000)	(0.000)
CV	Yes	Yes	Yes	Yes	Yes	Yes
City	Yes	Yes	Yes	Yes	Yes	Yes
Year	Yes	Yes	Yes	Yes	Yes	Yes
N	10980	10980	10980	10980	10980	10980
R2	0.847	0.848	0.055	0.118	0.078	0.016

Note: p-values in parentheses

* p < 0.1

** p < 0.05

*** p < 0.01.

## 5. Moderating effect analysis

This section investigates if the influence of air pollution on digital finance differs based on variations in urbanization levels, economic development stages, regional R&D levels, and urban salary scales. We grouped the samples based on the annual median, with the regression analysis results presented in Table 9.

**Table 9:** Moderating analysis.

	(1)	(2)	(3)	(4)	(5)	(6)	(7)	(8)
	Low urbanization	High urbanization	Underdeveloped	Developed	Low R&D	High R&D	Low salary	High salary
	DIF	DIF	DIF	DIF	DIF	DIF	DIF	DIF
$\hat{PM10}$	-1.120***	-0.345	-1.476***	-0.286	-1.405***	-0.037	-1.104***	-0.449
	(0.000)	(0.264)	(0.000)	(0.399)	(0.000)	(0.906)	(0.000)	(0.539)
lnManufacturer	0.043*	-0.025	0.005	-0.130***	0.014	-0.042**	0.067***	0.027
	(0.066)	(0.171)	(0.726)	(0.003)	(0.527)	(0.018)	(0.002)	(0.312)
FinancialLevel	0.035**	0.007	0.015	0.031	0.020	0.034*	0.018	0.114***
	(0.013)	(0.717)	(0.200)	(0.279)	(0.160)	(0.058)	(0.171)	(0.000)
lnGDP	-0.013	0.055***	0.058***	0.235***	0.043*	0.060***	-0.028	0.092***
	(0.638)	(0.002)	(0.000)	(0.000)	(0.085)	(0.001)	0.067***	0.027
CV	Yes	Yes	Yes	Yes	Yes	Yes	Yes	Yes
City	Yes	Yes	Yes	Yes	City	Yes	Yes	Yes
_cons	4.535***	5.206***	4.428***	3.900***	4.575***	4.942***	4.832***	3.964***
	(0.000)	(0.000)	(0.000)	(0.000)	(0.000)	(0.000)	(0.000)	(0.000)
Year	Yes	Yes	Yes	Yes	Yes	Yes	Yes	Yes
N	5810	5170	5963	5017	5589	5391	10228	752
R2	0.044	0.051	0.056	0.060	0.080	0.025	0.056	0.112

Note: p-values in parentheses

* p < 0.1

** p < 0.05

*** p < 0.01.

### 5.1 Moderating effect of urbanization

Urbanization in China places an emphasis on infrastructure development and the application of digital technologies. As digitalization progresses, it can boost the growth of internet-based and digital finance. Thus, cities with advanced urbanization might offset the adverse effects of air pollution on digital finance. To measure the level of urbanization, we utilize the proportion of the urban employed population relative to the overall employed population. Columns (1) and (2) of Table 9 indicate that while the detrimental effects of air pollution on digital finance are pronounced in regions with lower urbanization, such impacts are not statistically significant in areas with higher urbanization levels.

### 5.2 Moderating effect of economic development

In economically advanced regions, the abundance of job opportunities and elevated wage levels can offset the negative impacts of air pollution, potentially restricting population migration. We employ the city’s GDP as a representative measure of its level of economic development. Columns (3) and (4) of Table 9 show that in economically less developed areas, the coefficient of PM10 stands at -1.476, significant at the 1% level. However, in economically prosperous regions, the coefficients are insignificant.

### 5.3 Moderating effect of R&D

Concurrently, developed regions typically have higher R&D expenditure and better-developed infrastructure. High R&D spending can help attract and retain young professionals, thereby mitigating the detrimental influence of air pollution on digital finance. We adopt the total urban R&D expenditure as a measure for our moderating analysis. Columns (5) and (6) of Table 9 reveal that while air pollution significantly impedes digital finance growth in regions with lower salaries, this effect is not evident in areas with higher salaries.

### 5.4 Moderating effect of the salary level

In cities with higher salaries, the elevated standard of living can counterbalance the negative influence of air pollution on both population migration and digital finance. We employ the average salary of city enterprises as a representative measure for managerial salary levels. Columns (7) and (8) of Table 9 indicate that while air pollution significantly curtails the development of digital finance in lower-salary areas, this detrimental impact is less pronounced in regions with higher salaries.

## 6. Mechanism analysis

### 6.1 Mechanism: Aggravating population loss and aging problems

This study analyzes the effects of air pollution on the population structure by utilizing the logarithm of the total urban population, the elderly dependency ratio, and the proportion of the population aged 65 and above. Columns (1) and (5) of Table 10 show that the outflow of population (POPULATION) in northern China is significantly more pronounced than in the South. This reduction in population could emphasize the challenges associated with aging. To probe this issue further, we conducted additional regressions focusing on the elderly dependency ratio (DEPENDR) and the proportion of the population aged 65 and above (OLDER). The findings from Table 10 indicate that air pollution contributes to increases in both DEPENDR and OLDER, underscoring the notion that air pollution intensifies both population decline and aging trends.

**Table 10:** Mechanism test.

Panel A	(1)	(2)	(3)	(4)
	POPULATION	DEPENDR	OLDER	MQF
GAP	-.044***	0.046***	0.017***	-.014***
	(0.000)	(0.000)	(0.000)	(0.000)
lnManufacturer	0.005	-0.006	-0.004	-0.130***
	(0.698)	(0.640)	(0.756)	(0.003)
FinancialLevel	0.015	0.016	0.016	0.031
	(0.141)	(0.142)	(0.141)	(0.279)
lnGDP	0.047***	0.068***	0.070***	0.235***
	(0.001)	(0.000)	(0.000)	(0.000)
R-squared	0.1622	0.128	0.119	0.015
Panel B	(5)	(6)	(7)	(8)
	POPULATION	DEPENDR	OLDER	MQF
$\hat{PM10}$	-2.145***	0.125***	0.131***	-2.90***
	(0.000)	(0.000)	(0.000)	(0.000)
lnManufacturer	0.067***	0.027	0.067***	0.027
	(0.002)	(0.312)	(0.002)	(0.312)
FinancialLevel	0.018	0.114***	0.018	0.114***
	(0.171)	(0.000)	(0.171)	(0.000)
lnGDP	-0.028	0.092***	-0.028	0.092***
	(0.334)	(0.000)	(0.334)	(0.000)
R-squared	0.113	0.150	0.197	0.111

Note: p-values in parentheses

* p < 0.1

** p < 0.05

*** p < 0.01. Panel A shows that mechanism variables regress GAP in the first stage. Panel B shows the second stage RDD regression of mechanism variables with $\hat{PM10}$.

Air pollution adversely impacts human health, which in turn can enhance population decline and accelerate the aging process within urban populations. Drawing from the life cycle theory, even as the wealth of older residents grows, their demand for credit is likely to decrease. Additionally, financial institutions often impose age-related constraints on loan disbursement, which can exclude certain older demographics, further dampening credit demand [39]. Consequently, the challenges associated with an aging population can stifle the progression of digital finance.

### 6.2 Mechanism: Reducing human capital quality

Based on the study of Ref. [40], we employed the principal component analysis (PCA) to develop the human capital quality index (MQF) and subsequently performed a discontinuity regression analysis. As indicated in Column (4) of Table 10, the quality of human capital in enterprises in northern China is significantly lower than in the South. Furthermore, Column (8) of Table 10 reveals that air pollution substantially diminishes the quality of enterprise human capital.

Digital finance development is underpinned by technology, which requires the support of high-quality talent. The population decline resulting from air pollution also adversely impacts the quality of an enterprise’s human capital [41], thereby constraining the advancement of digital finance.

## 7. Conclusion and policy implications

### 7.1 Conclusion

This research comprehensively examines the influence of air pollution on the development of digital finance using data from 337 cities in China from 2013 to 2020. The results of our study emphasize the adverse impact that air pollution has on the development of digital finance. Interestingly, this effect is more noticeable in cities, distinguished by diminished degrees of urbanization, economic expansion, average salary, and research and development funding. Further analysis regarding the mechanisms showed that air pollution hinders the expansion of digital finance through the accelerated migration of the younger population. As a consequence, this demographic change causes a reconfiguration of the population as a whole, which ultimately diminishes the pool of skilled personnel that is critical for the development of digital finance. Moreover, the results of this study show that air pollution significantly diminishes the quality of enterprise human capital.

### 7.2 Policy implications

The findings of this research have significant implications for policymakers, particularly as they navigate the intertwined challenges of environmental sustainability and financial digitalization. First, the government should amplify its commitment to environmental stewardship. This involves introducing regulatory measures aimed at reducing air pollution. The government should introduce strategies that promote the adoption of cleaner energy sources, enhance the efficiency and reach of public transport systems, and ensure that firms responsible for pollution face significant economic penalties, such as fines or taxes. Furthermore, the study emphasizes the need for an intentional and targeted support system for the digital finance sector, especially in regions trailing in terms of urbanization, economic prowess, average wage benchmarks, and R&D initiatives. Governments could spur progress in these areas through a suite of interventions, offering fiscal incentives or subsidies to emerging digital finance ventures, amplifying efforts in education and training tailored for this sector, and bolstering the underlying financial infrastructure, all while maintaining rigorous oversight to ensure ethical practices. Lastly, the ripple effects of air pollution, notably its role in reshaping demographic dynamics and declining human capital, cannot be overlooked. Governments, familiar with these consequences, should be proactive in devising strategies to both attract and retain top-tier talent. This could mean elevating urban life quality by refining living standards, extending comprehensive social welfare programs and public services, and fostering a thriving job market with diverse opportunities. As the future unfolds, the balancing act between sustainable urban development and technological and financial advancement will be pivotal, and the insights from this research provide a roadmap for informed decision-making.

### 7.3 Limitations and future research direction

Although this research was extensive in its coverage of data from 337 Chinese cities, the study has some limitations. In this study, it is possible that the indices utilized do not entirely capture the intricacies of air pollution and digital finance, and future research studies can fill this gap by using different indices for digital finance. This study is focused on China and thus restricts the direct applicability of our findings to broader global contexts. Therefore, future studies can be carried out in the same context but in different countries and regions. Moreover, future research endeavors may consider extending the time scope, integrating primary data to improve the accuracy and comprehensiveness of the study, or expanding the geographical scope to examine whether the observed patterns persist in diverse socio-economic and environmental contexts.
